# *p*-Aminoacetophenonic Acids Produced by a Mangrove Endophyte *Streptomyces* sp. (strain HK10552)

**DOI:** 10.3390/molecules15042782

**Published:** 2010-04-16

**Authors:** Fangfang Wang, Minjuan Xu, Qingshan Li, Isable Sattler, Wenhan Lin

**Affiliations:** 1 State Key Laboratory of Natural and Biomimetic Drugs, Peking University, Beijing 100191, China; E-Mails: fangfang70303@yahoo.com.cn (F.F.W.); whlin@bjmu.edu.cn (W.L.); 2 School of Pharmaceutical Sciences, Shangxi Medical University, Shanxi 030001, China; 3 Shanghai Center for Systems Biomedicine, Shanghai Jiaotong University, Shanghai 200240, China; 4 Leibniz Institute for Natural Products Research and Infection Biology, Hans-Knöll Institute, Beutenbergstr.11a, D-07745 Jena, Germany; E-Mail: isattler@rhrk.uni-kl.de (I.S.)

**Keywords:** *p*-aminoacetophenonic acids, endophyte, mangrove plant, *Aegiceras corniculatum*

## Abstract

Four new *p*-aminoacetophenonic acids, named (2*E*)-11-(4′-aminophenyl)-5,9-dihydroxy-4,6,8-trimethyl-11-oxo-undec-2-enoic acid (**1**), 9-(4′-aminophenyl)-3,7-dihydroxy-2,4,6-trimethyl-9-oxo-nonoic acid (**2**), (2*E*)-11-(4′-aminophenyl)-5,9-*O*-cyclo-4,6,8-trimethyl-11-oxo-undec-2-enoic acid (**3**) and 9-(4′-aminophenyl)-3,7-*O*-cyclo-2,4,6-trimethyl-9-oxo-nonoic acid (**4**), were isolated from an endophyte *Streptomyces* sp. (strain HK10552) of the mangrove plant *Aegiceras corniculatum*. The structures of **1**–**4** were elucidated by using spectroscopic analyses. The relative stereoconfigurations of compounds **3** and **4** were determined by NOESY experiments. In the bioassay test, **1**–**4** showed no cytotoxicity against the Hela cell lines. Compound **4** also showed no inhibitory bioactivity on HCV protease and SecA ATPase and wasn’t active against VSVG/HIV-luc pseudotyping virus.

## 1. Introduction 

Mangrove endophytes, including actinomycetes and fungi, have been recognized as rich sources of structurally unique and biologically active secondary metabolites [1]. It was reported that 58.3% from 60 strains of endophytic fungi, isolated from mangrove plant *Aegiceras corniculatum, Excoecaria agallocha, Kandelia candel, Bruguiera gymnorrhiza, Avicennia mariana, Heritiera littoralis*, have antibacterial activities and 81.7% strains have antifungal activities [2]. 

In general, the production of secondary metabolites is potentially useful for pharmaceutical and agricultural applications [3]. Our previous investigation of endophytic microorganisms from mangrove plants resulted in a number of bioactive and structurally unique metabolites: new pyrrole and indole alkaloids from an endophytic *Fusarium incarnatum* isolated from *A. corniculatum* [4], new cyclopentenone derivatives from another endophytic *Streptomyces* sp. [5], indole triterpenoids [6], polyketides [7], and penicillenols [8] were isolated from endophytic *Penicillium* sp. associated with *A. corniculatum.* The indole triterpenoids showed blocking activity on large-conductance calcium-activated potassium channels [6,9]. 

**Figure 1:**
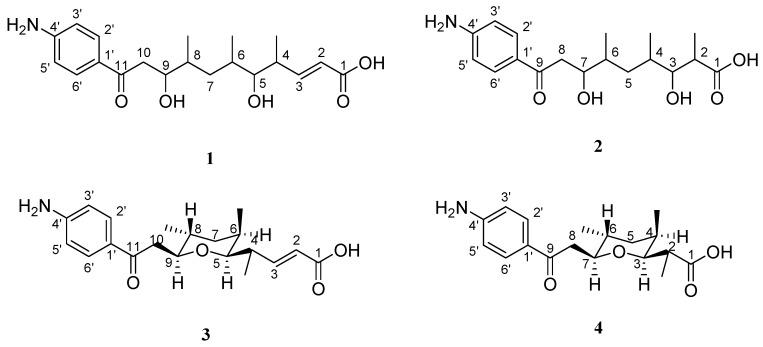
New *p*-aminoacetophenonic acids **1–4**.

In the course of ongoing investigations on natural products for the metabolic relationship between mangrove plants and their endophytes, an actinomyces (strain HK10552) was isolated from the leaves of *A. corniculatum* (Aegicerataceae). This strain produced four new *p*-aminoacetophenonic acids, whose derivatives were previously isolated from a mangrove endophyte *Streptomyces griseus* subsp. [10], namely (2*E*)-11-(4′-aminophenyl)-5,9-dihydroxy-4,6,8-trimethyl-11-oxo-undec-2-enoic acid (**1**), 9-(4′-aminophenyl)-3,7-dihydroxy-2,4,6-trimethyl-9-oxo-nonoic acid (**2**), (2*E*)-11-(4′-aminophenyl)-5,9-*O*-cyclo-4,6,8-trimethyl-11-oxo-undec-2-enoic acid (**3**) and 9-(4′-aminophenyl)-3,7-*O*-cyclo-2,4,6-trimethyl-9-oxo-nonoic acid (**4**) (Figure 1), along with several known compounds, like 4-methylamino-benzoic acid, methyl-2-(2-hydroxyphenyl)acetate, 4-hydroxybenzoic acid, (2*E*)-3-(4-hydroxy-3-methoxyphenyl)-2-propenoic acid, methyl-2-(1H-indol-3-yl)acetate, (2*E*)-(4-hydroxyphenyl)-2-propenoic acid, (2*E*)-(4-hydroxyphenyl)-2-propenoic acid, 1H-indole-3-acetic acid, indole-3-carboxylic acid, cyclo(phenylalanyl-seryl)-3,3-bis(30-indolyl)propane-1,2-diol and 2-hydroxybenzeneacetic acid. In this paper, we report the structural elucidation of the new *p*-aminoacetophenonic acids **1–4**.

## 2. Results and Discussion

**Figure 2:**
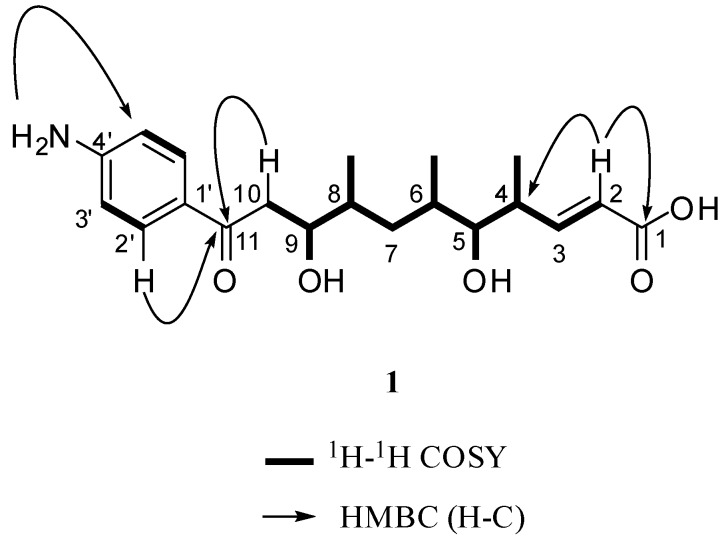
Key COSY and HMBC correlations of compound **1**.

Compound **1** was isolated as a yellow oil and its molecular formula was determined to be C_20_H_29_NO_5_ on the basis of HRESIMS analysis (364.21266 [M+H]^+^, calcd. for C_20_H_30_NO_5_, *m*/*z* 364.21185), which indicated seven degrees of unsaturation. In the ^1^H NMR spectrum three methyl groups at δ_H_ 0.76 (d, *J* = 6.36 Hz), 0.80 (d, *J* = 6.55 Hz) and 0.97 (d, *J* = 6.32 Hz), and two exchangeable protons at *δ* 4.35 (brs, OH-9) and 4.54 (brs, OH-5) were detected (See Table 1). Carbon signals of the ^13^C-NMR spectrum at δ*_C_* 153.9 and 168.0 indicated an olefinic bond, which was supported by the correlation signal of two relevant protons at δ 5.73 (1H, d, *J* = 15.73 Hz) and δ 6.85 (1H, m) in the ^1^H-^1^H COSY spectrum (see Figure 2.). This olefinic bond was assigned to be *E*-configurated on the basis of its large vicinal coupling constant of 15.73 Hz [11]. The substituted position of the double bond in the aliphatic chain was deduced by the correlation from H-2 to C-4 in HMBC spectrum and the correlation signal between H-3 and H-4 in ^1^H-^1^H COSY spectrum. An attached carboxylic acid group at the double bond was assigned on the basis of the high-field carboxy signal at δ 168.0 and the HMBC correlation from H-2 to C-1. Quarternary carbon signals at δ 197.3 and 168.0 implied the presence of keto-carbonyl acid groups. The ^1^H-^1^H COSY correlations confirmed the assembly of the aliphatic chain between H-2 and H_2_-10 and the position of Me-4, OH-5, Me-6, Me-8 and OH-9. Aromatic proton signals at δ 6.55 (2H, d, *J* = 8.41 Hz), 7.68 (2H, d, *J* = 8.42 Hz) were attributed to a *p-*disubstituted phenyl moiety, the assignment of which was supported by carbon signals at δ 125.7 (C-1′), 131.0 (C-2′), 112.9 (C-3′), and 153.9 (C-4′) in the ^13C^-NMR spectrum. One of the substituted groups of the aromatic ring was 4´-NH_2_ (6.02, s), which was deduced from the HMBC correlation between C-3´ and 4´-NH_2_ (6.02, s) and the aminophenonic moiety was linked to C-11 as deduced from the HMBC correlation between H-2´ and C-11 (see Figure 2). Thus, compound **1** was determined as (2*E*)-11-(4-aminophenyl)-5,9-dihydroxy-4,6,8-trimethyl-11-oxo-undec-2-enoic acid (**1**).

Compound **2** was isolated as a yellow oil. The determination of its molecular formula C_18_H_27_NO_5_ was based on HRESIMS analysis (338.19701 [M+H]^+^, calcd. for C_20_H_30_NO_5_, *m*/*z* 338.19620), which indicated the molecule with six degrees of unsaturation. Comparison of ^1^H- and ^13^C-NMR data of **2** with those of **1** showed that **2** was a homologue of **1** with the olefinic bond missing. The newly found HMBC correlations from H-2 (2.36, m) to C-1 (176.5, s) confirmed the suggested structure. Therefore, **2** was elucidated to be 9-(4-aminophenyl)-3,7-dihydroxy-2,4,6-trimethyl-9-oxo-nonoic acid (**2**).

**Figure 3:**
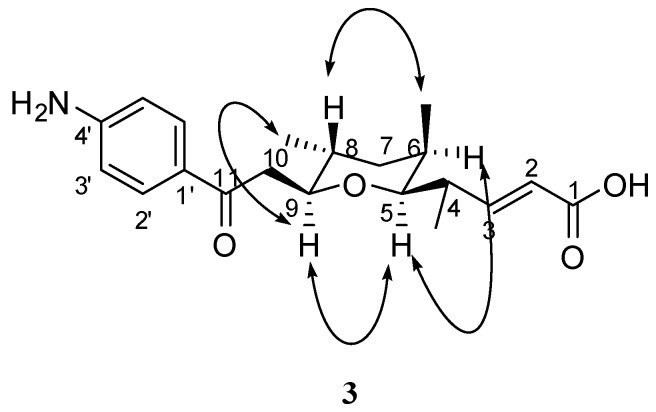
Key NOE correlations of compound **3**.

Compound **3**, a yellow oil, was determined to be C_20_H_27_NO_4_ on the basis of its HRESIMS data (346.20109 [M+H]^+^, calcd for C_20_H_28_NO_4_, *m*/*z* 346.20128), requiring eight degrees of unsaturation. According to the comparison of mass spectral data between **3** and **1**, there is 18 amu missing suggesting that **3** was the product of dehydration-condensation from **1**. Compound **3** contained similar structural elements as **1**, expect for the two missing hydroxyl groups, as observed in the ^1^H-NMR spectrum. The carbon signals at δ 29.3 (d, C-6), 30.2 (d, C-8) were shifted remarkably to highfield by 4–8 ppm as compared with **1**, while signals at δ 82.8 (d, C-5), 81.7 (d, C-9) were moved downfield for 6–9 ppm, indicating that the attached two hydroxyl groups of **1** were condensed to form a pyran-ring in **3**. The HMBC correlations from H-5 (3.06, d, *J* = 9.62 Hz) to C-9 (81.7, d) confirmed this finding. The relative stereoconfiguration of **3** was determined on the basis of as NOESY experiment. NOE effects were observed between H-5/H-6, H-5/H-9 and Me-8/H-9 (see Figure 3) indicating that H-5, H-6, H-9, Me-8 were on the same side of the ring with *α*-orientation, and in turn H-8 and Me-6 were assumed to be in *β*-orientation confirmed by an NOE correlation between H-8 and Me-6. Thus, compound **3** was (2*E*)-11-(4′-aminophenyl)-5,9-*O*-cyclo-4,6,8-trimethyl-11-oxo-undec-2-enoic acid (**3**).

Compound **4**, was also isolated as a yellow oil. Its molecular formula was determined to be C_18_H_25_NO_4_ on the basis of its HRESIMS data (320.18587 [M+H]^+^, calcd for C_20_H_28_NO_4_, *m*/*z* 320.18563) requiring seven degrees of unsaturation. ^1^H and ^13^C NMR data were suggesting that compound **4** held a similar structure as that of **3**, except for a missing double bond, as observed in the ^1^H NMR spectrum. HMBC correlations between H-3 (3.45, m) and C-7 (81.0, d) and C-1 (176.7, s) confirmed this finding. The relative stereoconfiguration of **4** was determined on the basis of an NOESY experiment. NOE correlations of H-4/H-7, H-4/H-3, H-6/Me-4, and Me-6/H-3, Me-6/H-7 indicated that H-3, H-4, H-7, Me-6 were located on the same side of the ring and suggesting to have a *α*-orientation, and in turn H-6 and Me-4 were assumed to be in the *β*-orientation. Thus, compound **4** was 9-(4′-aminophenyl)-3,7-*O*-cyclo-2,4,6-trimethyl-9-oxo-nonoic acid (**4**).

In cytotoxicity testing against Hela cells compounds **1–4** showed no cytotoxicity (IC_50_ > 100 μg/mL). In further testing, compound **4** proved to be inactive against HCV protease and SecA ATPase and did not inhibit VSVG/HIV-luc pseudotyping virus on 293T cell line.

Compounds **1** and **2** show certain structural similarity to the polyene antibiotics, e.g. candicidin produced by *Streptomyces griseus*, which consists of the aromatic moiety, the macrolide ring and the amino sugar moiety. Compounds **1** and **2** have different polyketide chain lengths which may be due to different biosynthetic polyketide assembly procedures. It was reported the the *p*-aminoacetophenone moiety of candicidin is synthesized from chorismic acid via the aromatic amino acid pathway and *p*-aminobenzoic acid (PABA) has been identified as the immediate precursor [12]. The isolation of 4-methylaminobenzoic acid from the crude extract in our study suggested the presence of this biosynthetic pathway *Streptomyces* sp. (strain HK10552). 

Polyene macrolide antibiotics are the most effective antifungal agents due to their potent broad spectrum fungicidal activity and relatively low frequency of resistance among the fungal pathogens [13]. Therefore, the novel derivatives of polyene antibiotics discovered in our study would be a potential target for further chemical and microbiological investigations in this respect. 

## 3. Experimental 

### 3.1. General

^1^H- and ^13C^-NMR spectra were measured on a Bruker Avance DRX 500 spectrometer using TMS as an internal standard. Chemical shifts (δ) expressed in parts per million (ppm) and coupling constants (*J*) are reported in Hertz (Hz). Optical rotations were recorded on a Perkin-Elmer 341 LC polarimeter. ESIMS spectra were measured on a Quattro. Premier XE tandem mass spectrometer (Micromass, UK), while HRESIMS spectra were measured on a LTQ Orbitrap X1 Thermo Scientific and a FT-MS-Bruker APEX IV (7.0T).Column chromatography was carried with silica gel (200–300 mesh), and GF_254_ Silica gel for TLC was provided by Qingdao Marine Chemistry Co.. Semipreparative HPLC was performed on an Alltech-HPLC (USA) using a Kromasil column (ODS, 10 μm, 10 × 250 mm). The chemical reagents used for chromatography were purchased from Beijing Chemical Works Co. Ltd. (Beijing).

### 3.2. Plant Material 

Leaves of *A. corniculatum* (Aegicerataceae) were collected near Xiamen City of Fujian Province, People’s Republic of China, in August 2002, and identified by Prof. Peng Lin of Xiamen University. Samples were deposited in the State Key Laboratory of Natural and Biomimetic Drugs, Peking University (No.200208082-2). Microbial materials were isolated from the leaves of the plant. The *Streptomyces* sp. (strain HK10552), subject of this study, was deposited in Hans-Knöll-Institute, Jena, Germany.

### 3.3. Strain Isolation, Characterization, and Cultivation

The strain was isolated from the mangrove plant *A. corniculatum.* Pieces of the leaves were rinsed with sterile water, sterilized by soaking in 70% ethanol (1 min) and 3.5% NaCl solution (3 min), and rinsed again with sterile water. Tiny pieces obtained by cutting with a sterile knife were placed on agar plates with GPY agar, which was supplemented with streptomycin (0.1 g/L) and chloramphenicol (0.2 g/L), and incubated at 22 ºC until growth appeared. Colonies were transferred to fresh agar plates for further growth. General laboratory cultivation was performed on malt agar [malt extract 20.0 g/L, yeast extract 2.0 g/L, glucose 10.0 g/L, (NH_4_)_2_HPO_4_ 0.5 g/L, pH 6.0] or the respective liquid medium at 22 ºC. For long-term preservation, cultures grown on agar plates supplemented with 5% glycerol were maintained in the vapor phase of liquid nitrogen. The strain isolate has been deposited in the strain collection of the Leibniz Institute for Natural Products Research and Infection Biology. 

For screening purposes, the strain was grown in 250 mL Erlenmeyer flasks with 100 mL of the production culture medium, consisting of saccharose 20 g/L, soybean flour10 g/L, cornsteep 10 g/L, and KCl 8 g/L, adjusted to pH 6.5 prior to sterilization. It was inoculated with pieces (1 × 1 cm^2^ from an agar plate of a well grown culture and cultivated as resting culture for 17 days at 22 ºC. The production culture was carried out in a 300 L fermentor filled with 200L of the above production medium; it was grown for 10 days at 22 ºC (aeration 50 L/min, pH 6.5) and stirred at 125 rpm. The inoculum (2 L) was obtained after several steps of resting cultures with increasing cultivation volume in a modified malt extract medium [malt extract 10.0 g/L, yeast extract 4.0 g/L, glucose 4.0 g/L, (NH_4_)_2_ HPO_4_ 0.5 g/L, pH 5.5].

### 3.4. Extraction and Isolation

Fermentation supernatant of *Streptomyces* sp. (strain HK10552) was filtered by centrifugation and subjected to a XZD-161M resin column (20 × 20 cm) eluted with MeOH/H_2_O (gradient from 40:60 to 90:10 in 38 min). Seven fractions were collected and lyophilized. Fractions 5-6 (total 2g) was further purified on Sephadex LH-20 eluted with MeOH (900 mL) to give six subfractions. Subfraction 6 (900 mg) was separated on a RP-18 silica gel column eluted with MeOH/H2O (gradient from 10:90 to 88:12, 1800 mL) to give six fractions (T1-T6). Fraction T2 (88.6 mg) and T3 (30.2 mg) was purified by semi-preparative HPLC with MeOH–H_2_O (1:1) as a mobile phase to yield compound **1** (13.9 mg) and **2** (19.6 mg). T4 (119.8mg) and T5 (88.2mg) was followed by the same way as T2 with MeOH–H_2_O (7:3) as a mobile phase to obtain **3** (29.7 mg) and **4** (40.9 mg).

*(2E)-11-(4-Aminophenyl)-5,9-dihydroxy-4,6,8-trimethyl-11-oxo-undec-2-enoic acid* (**1**). Yellow oil; ${\left[\alpha \right]}_{D}^{22}$ -20.0º (*c* 0.015, MeOH); UV *λ _max_* (MeOH) 318, 206 nm; IR (KBr) *ν_max_* 3,733, 3,357, 2,964, 2,931, 1,698, 1,650, 1,595, 1,379, 1,175, 826 cm^-1^; ^1^H- and ^13^C-NMR data, see Table 1; ESIMS *m*/*z* positive 364.4 [M + H]^+^; HRESIMS *m*/*z* 364.21266 [M + H]^+^ (calcd for C_20_H_30_NO_5_, *m*/*z* 364.21185).

*9-(4-Aminophenyl)-3,7-dihydroxy-2,4,6-trimethyl-9-oxo-nonoic acid* (**2**). Yellow oil; ${\left[\alpha \right]}_{D}^{22}$ +1.35º (*c* 0.07, MeOH); UV *λ*_max_ (MeOH) 319, 231, 195 nm; IR (KBr) *ν*_max _ 3,733, 3,515, 3,446, 3,354, 2,961, 2,933, 2,869, 1,772, 1,651, 1,629, 1,179, 1,592, 830 cm^-1^; ^1^H- and ^13^C-NMR data, see Table 1; ESIMS *m*/*z* positive 338.5 [M + H]^+^; HRESIMS *m*/*z* 338.19701 [M + H]^+^ (calcd for C_18_H_28_NO_5_, *m*/*z* 338.19620).

*(2E)-11-(4′-aminophenyl)-5,9-O-cyclo-4,6,8-trimethyl-11-oxo-undec-2-enoic acid* (**3**). Yellow oil; ${\left[\alpha \right]}_{D}^{22}$ -35.48º (*c* 0.03, MeOH); UV *λ*_max_ (MeOH) 314, 207 nm; IR (KBr) *ν*_max_ 3,448, 3,356, 3,230, 2,964, 2,911, 1,700, 1,652, 1,596, 1,381, 1,025, 1,051, 828 cm^-1^; ^1^H- and ^13^C-NMR data, see Table 1; ESIMS *m*/*z* positive 346.6 [M + H]^+^; HRESIMS *m*/*z* 346.20109 [M + H]^+^ (calcd for C_20_H_28_NO_4_, *m*/*z* 346.20128).

*9-(4′-aminophenyl)-3,7-O-cyclo-2,4,6-trimethyl-9-oxo-nonoic acid* (**4**). Yellow oil; ${\left[\alpha \right]}_{D}^{22}$ -3.66º (*c* 0.04, MeOH); UV *λ*_max_ (MeOH) 316, 230,192 nm; IR (KBr) *ν*_max_ 3,448, 3,358, 3,238, 2,923, 1,720, 1,636, 1,596, 1,379, 1,025, 828 cm^-1^; ^1^H- and ^13^C-NMR data, see Table 1; ESIMS *m*/*z* positive 320.4 [M + H]^+^; HRESIMS *m*/*z* 320.18587 [M + H]^+^ (calcd for C_18_H_26_NO_4_, *m*/*z* 320.18563).

## 4. Conclusions 

Isolation of four new *p*-aminoacetophenonic acids from an endophyte *Streptomyces* sp. (strain HK10552) of the mangrove plant *A. corniculatum* was reported. These compounds were identified as (2*E*)-11-(4′-aminophenyl)-5,9-dihydroxy-4,6,8-trimethyl-11-oxo-undec-2-enoic acid (**1**), 9-(4′-amino-phenyl)-3,7-dihydroxy-2,4,6-trimethyl-9-oxo-nonoic acid (**2**), (2*E*)-11-(4′-aminophenyl)-5,9-*O*-cyclo-4,6,8-trimethyl-11-oxo-undec-2-enoic acid (**3**) and 9-(4′-aminophenyl)-3,7-*O*-cyclo-2,4,6-trimethyl-9-oxo-nonoic acid (**4**). In the bioassay test, **1–4** showed no cytotoxicity against the Hela cell lines. Compound **4** also showed no inhibitory bioactivity on HCV protease and SecA ATPase and was not active against VSVG/HIV-luc pseudotyping virus. Further chemical and pharmaceutical investigation will be carried on in order to explore the rich source of bioactive secondary metabolites of mangrove endophytes.

## Figures and Tables

**Table 1:** ^1^H- and ^13^C-NMR Data of Compounds **1**, **2**, **3** and **4**. ^a^

Position	1		2		3		4	
	*δ_H_*	*δ_C_*	*δ_H_*	*δ_C_*	*δ_H_*	*δ_C_*	*δ_H_*	*δ_C_*
1-COOH	--	168.0 s	--	176.5 s	--	167.8 s	--	176.7 s
2	5.73 d 15.73	122.0 d	2.36 m	44.2 d	5.59 d 15.79	121.2 d	2.20 m	42.6 d
3	6.85 m	152.0 d	3.42 d 8.44	76.0 d	6.75 dd 6.91 15.78	152.7 d	3.45 m	81.3 d
4	--	39. 6 d	1.60 m	31.8 d	2.20 m	38.0 d	1.76 m	28.2d
5	3.06 m	77.9 d	1.10 m 1.40 m	36.8 t	3.06 d 9.62	82.8 d	1.58 m 1.40 m	40.0 t
6	1.45 m	33.7 d	1.60 m	36.3 d	1.78 m	29.3 d	1.58 m	30.3 d
7	1.09 m 1.22 m	35.8 t	3.90 m	71.8 d	1.38 m 1.59 m	40.0 t	3.46 m	81.0 d
8	1.56 m	36.3 d	2.51 m 2.82 dd 8.81 15.03	41.3 t	--	30.2 d	2.93 dd 3.91 15.73 2.84 dd 6.20 15.70	42.9 t
9	3.86 m	72.2 d	--	197.3 s	3.41 m	81.7 d	--	195.6 s
10	2.68 dd 2.78 14.93 2.88 dd 8.65 15.06	41.7 t	--	--	2.83 dd 7.60 15.00 2.97 dd 3.55 15.10	42.5 t	--	--
11	--	197.3 s	--	--	--	196.1 s	--	--
2-Me	--	--	0.96 d 6.91	14.6 q	--	--	0.92 d 6.90	13.5 q
4-Me	0.97 d 6.32	17.7 q	0.71 d 6.48	12.5 q	0.84 m	15.2 q	0.73 d 6.10	12.1 q
6-Me	0.76 d 6.36	14.3 q	0.88 m	15.6 q	0.97 m	12.3 q	0.84 q 6.85	18.1 q
8-Me	0.80 d 6.55	15.6 q	--	--	0.76 d 6.14	18.0 q	--	--
1′	--	125.7 s	--	125.7 s	--	125.9 s	--	125.6 s
2′	7.73 d 8.42	131.0 d	7.68 d 8.28	131.0 d	7.63 d 8.34	131.0 d	7.65 d 8.34	131.0 d
3′	6.55 d 8.41	112.9 d	6.55 d 8.28	112.9 d	6.52 d 8.34	112.9 d	6.54 d 8.32	112.9 d
4′	--	153.9 s	--	153.9 s	--	153.7s	--	153.8 s
4′-NH_2_	6.02 s	--	6.02 s	--	5.94 s	--	5.98 s	--
5′	7.68 d 8.42	131.0 d	7.68 d 8.28	131.0 d	7.63 d 8.34	131.0 d	7.65 d 8.34	131.0 d
6′	6.55 d 8.41	112.9 d	6.55 d 8.28	112.9 d	6.52 d 8.34	112.9 d	6.54 d 8.32	112.9 d
3-OH	--	--	4.43 brs	--	--	--	--	--
5-OH	4.54 brs	--	--	--	--	--	--	--
7-OH	--	--	4.43 brs	--	--	--	--	--
9-OH	4.35 brs	--	--	--	--	--	--	--

^a^ Measured in DMSO-*d_6_*, Chemical shifts (*δ*) in ppm.
